# Supplementary material for: Association between pan-immune-inflammation value and clinical outcomes in critically ill patients with hyperlipidemia: An observational study
Source: PLoS One. 2026 Jun 1;21(6):e0349954. doi: 10.1371/journal.pone.0349954 (PMC13225374; doi:10.1371/journal.pone.0349954)
Supplement: S1 Table — Baseline characteristics of critically ill patients with hyperlipidemia stratified by lnPIV quartiles. (DOCX) [file pone.0349954.s001.docx]

**Supplementary Table S1:** Group differences by lnPIV quartiles.

| Categories | Q1 (N=3102） | Q2 (N=3102） | Q3 (N=3102） | Q4 (N=3102） | P-value |
| --- | --- | --- | --- | --- | --- |
| Age(years) | 69.2 (11.3) | 69.8(11.6) | 70.6(12.4) | 71.5(12.6) | <0.001 |
| Male, n(%) | 1096(35.3) | 1026(33.1) | 1135(36.6) | 1299(41.9) | <0.001 |
| White, n(%) | 2056(66.3) | 2161(69.7) | 2115(68.2) | 2113(68.1) | 0.042 |
| Vital signs |  |  |  |  |  |
| Heartrate, beats/min | 80.0[73.0,87.0] | 80.0[72.0,89.0] | 84.0[74.0,97.0] | 91.0[79.0,105.0] | <0.001 |
| MBP, mmHg | 78.0[69.0,87.0] | 80.0[71.0,91.0] | 81.0[71.0,93.0] | 82.0[71.0,94.0] | <0.001 |
| Respiratory rate, beats/min | 16.0[14.0,18.5] | 16.0[14.5,20.0] | 18.0[15.0,22.0] | 20.0[17.0,25.0] | <0.001 |
| Laboratory tests |  |  |  |  |  |
| Sodium(mEq/L) | 138.0[136.0,141.0] | 139.0[136.0,141.0] | 138.0[136.0,141.0] | 138.0[135.0,141.0] | <0.001 |
| Potassium(mEq/L) | 4.2[3.9,4.6] | 4.2[3.9,4.6] | 4.2[3.8,4.6] | 4.2[3.8,4.7] | <0.001 |
| Chloride(mEq/L) | 108.0[105.0,110.0] | 107.0[103.0,109.0] | 105.0[101.0,108.0] | 102.0[98.0,107.0] | <0.001 |
| Bicarbonate(mEq/L) | 23.0[21.0,24.0] | 23.0[21.0,25.0] | 23.0[20.0,25.0] | 22.0[19.0,25.0] | <0.001 |
| Anion gap(mEq/L) | 12.0[10.0,14.0] | 12.0[10.0,15.0] | 14.0[11.0,16.0] | 15.0[13.0,18.0] | <0.001 |
| BUN(mg/dL) | 16.0[12.3,23.0] | 17.0[13.0,26.0] | 20.0[14.0,32.0] | 25.0[17.0,41.0] | <0.001 |
| Creatinine(mg/dL) | 0.9[0.7,1.2] | 0.9[0.8,1.2] | 1.0[0.8,1.5] | 1.2[0.8,1.9] | <0.001 |
| Glucose(mg/dL) | 120.0[105.0,141.0] | 123.0[105.0,148.0] | 130.0[109.0,166.0] | 143.0[112.0,192.0] | <0.001 |
| HbA1c(%) | 5.9[5.5, 6.7] | 5.9[5.5,6.7] | 5.9[5.5,6.8] | 5.9[5.5,6.7] | 0.014 |
| Comorbidities |  |  |  |  |  |
| CHF(%) | 766(24.7) | 917(29.6) | 1134(36.6) | 1295(41.7) | <0.001 |
| Atrial fibrillation(%) | 1118(36.0) | 1188(38.3) | 1193(38.5) | 1189(38.3) | 0.148 |
| Hypertension(%) | 1829(59.0) | 1757(56.6) | 1639(52.8) | 1478(47.6) | <0.001 |
| Cerebrovascular disease(%) | 386(12.4) | 474(15.3) | 548(17.7) | 472(15.2) | <0.001 |
| COPD(%) | 660(21.3) | 640(20.6) | 825(26.6) | 972(31.3) | <0.001 |
| Liver disease(%) | 236(7.6) | 220(7.1) | 246(7.9) | 262(8.4) | 0.241 |
| Renal disease(%) | 655(21.1) | 691(22.3) | 813(26.2) | 906(29.2) | <0.001 |
| Diabetes(%) | 1222(39.4) | 1272(41.0) | 1337(43.1) | 1338(43.1) | 0.006 |
| Malignant tumor(%) | 334(10.8) | 237(7.6) | 320(10.3) | 511(16.5) | <0.001 |
| Medications |  |  |  |  |  |
| Norepinephrine(%) | 482(15.5) | 525(16.9) | 715(23.0) | 1000(32.2) | <0.001 |
| Glucocorticoid (%) | 500(16.1) | 506(16.3) | 713(23.0) | 933(30.1) | <0.001 |
| Statin(%) | 2452(79.0) | 2484(80.1) | 2321(74.8) | 2195(70.8) | <0.001 |
| SOFA | 2.0[1.0,4.0] | 2.0[0.0,4.0] | 1.0[0.0,3.0] | 1.0[0.0,3.0] | <0.001 |

Baseline characteristics of critically ill patients with hyperlipidemia stratified by lnPIV quartiles. Continuous variables are presented as mean (standard deviation) or median (interquartile range), and categorical variables are shown as count (percentage). Key metrics and scores included are: MAP, mean arterial pressure; BUN, blood urea nitrogen; CHF, congestive heart failure; COPD, chronic obstructive pulmonary disease; SOFA, Sequential Organ Failure Assessment; PIV, pan-immune-inflammation value.
