# Supplementary material for: Association between pan-immune-inflammation value and clinical outcomes in critically ill patients with hyperlipidemia: An observational study
Source: PLoS One. 2026 Jun 1;21(6):e0349954. doi: 10.1371/journal.pone.0349954 (PMC13225374; doi:10.1371/journal.pone.0349954)
Supplement: S2 Table — (DOCX) [file pone.0349954.s002.docx]

**Supplementary Table S2:** Predictive performance of PIV and individual blood cell parameters for in-hospital mortality.

|  | Cutoff | AUC (95% CI) | Sensitivity | Specificity | Youden Index |
| --- | --- | --- | --- | --- | --- |
| lnPIV | 7.06 | 0.664 (0.648–0.681) | 0.529 | 0.716 | 0.246 |
| Neutrophils | 13.35 | 0.578 (0.559–0.596) | 0.302 | 0.829 | 0.131 |
| Monocytes | 0.64 | 0.571 (0.552–0.589) | 0.515 | 0.608 | 0.123 |
| Platelets | 293.50 | 0.490 (0.470–0.509) | 0.180 | 0.870 | 0.050 |
| 1/Lymphocytes | 0.95 | 0.662 (0.646–0.678) | 0.583 | 0.672 | 0.255 |

AUC, area under the receiver operating characteristic curve; CI, confidence interval. Cutoff values were determined by maximizing the Youden index.
