# Supplementary material for: Association between pan-immune-inflammation value and clinical outcomes in critically ill patients with hyperlipidemia: An observational study
Source: PLoS One. 2026 Jun 1;21(6):e0349954. doi: 10.1371/journal.pone.0349954 (PMC13225374; doi:10.1371/journal.pone.0349954)
Supplement: S3 Table — (DOCX) [file pone.0349954.s003.docx]

**Supplementary Table S3:** Predictive performance of PIV and other inflammatory markers for in-hospital mortality.

|  | Cutoff | AUC (95% CI) | Sensitivity | Specificity | Youden Index |
| --- | --- | --- | --- | --- | --- |
| lnPIV | 7.06 | 0.664 (0.648–0.681) | 0.529 | 0.716 | 0.246 |
| NLR | 8.505 | 0.687 (0.670–0.703) | 0.621 | 0.687 | 0.308 |
| PLR | 177.865 | 0.633 (0.615–0.651) | 0.588 | 0.640 | 0.228 |
| SII | 2153.835 | 0.651 (0.634–0.668) | 0.477 | 0.765 | 0.242 |
| MLR | 0.476 | 0.682 (0.666–0.698) | 0.684 | 0.594 | 0.278 |

NLR, neutrophil-to-lymphocyte ratio; PLR, platelet-to-lymphocyte ratio; SII, systemic immune-inflammation index; MLR, monocyte-to-lymphocyte ratio; SIRI, systemic inflammation response index.
