# Supplementary material for: Association between pan-immune-inflammation value and clinical outcomes in critically ill patients with hyperlipidemia: An observational study
Source: PLoS One. 2026 Jun 1;21(6):e0349954. doi: 10.1371/journal.pone.0349954 (PMC13225374; doi:10.1371/journal.pone.0349954)
Supplement: S4 Table — (DOCX) [file pone.0349954.s004.docx]

**Supplementary Table S4:** Improvement in predictive performance by adding inflammatory markers to the SOFA score.

| Model Comparison | SOFA Alone AUC | SOFA + Marker AUC | ΔAUC | Z Value | P Value |
| --- | --- | --- | --- | --- | --- |
| SOFA vs SOFA+lnPIV | 0.551 | 0.667 | +0.116 | -10.867 | < 0.001 |
| SOFA vs SOFA+NLR | 0.551 | 0.670 | +0.119 | -14.984 | < 0.001 |
| SOFA vs SOFA+PLR | 0.551 | 0.635 | +0.084 | -11.751 | < 0.001 |
| SOFA vs SOFA+SII | 0.551 | 0.654 | +0.103 | -12.966 | < 0.001 |
| SOFA vs SOFA+MLR | 0.551 | 0.675 | +0.124 | -14.179 | < 0.001 |
| SOFA vs SOFA+SIRI | 0.551 | 0.673 | +0.122 | -13.795 | < 0.001 |

ΔAUC, difference in AUC between SOFA alone and SOFA combined with each marker. P values were calculated using the DeLong test.
