# Supplementary material for: Association between pan-immune-inflammation value and clinical outcomes in critically ill patients with hyperlipidemia: An observational study
Source: PLoS One. 2026 Jun 1;21(6):e0349954. doi: 10.1371/journal.pone.0349954 (PMC13225374; doi:10.1371/journal.pone.0349954)
Supplement: Attachment — Submitted filename: Response to Reviewers.docx [file pone.0349954.s008.docx]

**Response to Reviewers**

Manuscript ID: PONE-D-25-56394

Title: Association between Pan-immune-inflammation value and clinical outcomes in critically ill patients with hyperlipidemia: An observational study

Dear Editor and Reviewers,

We sincerely thank the editor and reviewers for their valuable time and insightful comments on our manuscript. We have carefully addressed each point raised and revised the manuscript accordingly. Below is our point-by-point response following the order of the decision letter.

Responses to Editorial Comments

Comment 1: PLOS ONE style requirements

Please ensure that your manuscript meets PLOS ONE's style requirements

Response:We have carefully reviewed the PLOS ONE formatting guidelines and revised the manuscript accordingly.

Comment 2: Author information

Please amend the manuscript submission data to include author Xiang-Zheng.

Response:We have updated the manuscript submission data to include author Xiang-Zheng as a co-author.

Comment 3: Supporting Information captions

Please include captions for your Supporting Information files at the end of your manuscript.

Response: We have added detailed captions for all Supporting Information files at the end of the main manuscript.

---

Responses to Journal Questions

Question 1: Is the manuscript technically sound, and do the data support the conclusions?

Reviewer #1: Partly

Reviewer #2: Yes

Reviewer #3: Yes

Response: We thank the reviewers for their assessment. To address Reviewer #1's concerns, we have made substantial revisions including: (1) adding HbA1c as a covariate, (2) adjusting for glucocorticoid use, and (3) demonstrating the incremental value of PIV beyond SOFA. These changes strengthen the technical soundness of our manuscript.

Question 2: Has the statistical analysis been performed appropriately and rigorously?

Reviewer #1: Yes

Reviewer #2: Yes

Reviewer #3: Yes

Response: We appreciate the reviewers' positive assessment of our statistical analyses. We have further enhanced the rigor by adding sensitivity analyses, multiple imputation details, and incremental value assessments.

Question 3: Have the authors made all data underlying the findings in their manuscript fully available?

Reviewer #1: No

Reviewer #2: No

Reviewer #3: Yes

Response: We have now made all data and code publicly available. The GitHub repository (https://github.com/huangxiaoying5676/PIV-hyperlipidemia-analysis) contains:

- Complete R analysis scripts

- Aggregated datasets (cleaned and imputed data)

- README file with replication instructions

---

\## Responses to Reviewer #1's Specific Comments

Comment 1.1: Since all of the patients in this study were critically ill, the title should include "critically ill patients with hyperlipidemia."

Response: We thank the reviewer for this suggestion. We have revised the title to: "Association between Pan-immune-inflammation value and clinical outcomes in critically ill patients with hyperlipidemia: An observational study." This change has been made on the title page (Page 1,Title).

Comment 1.2:Although blood glucose levels are used in model 3.HbA1c is likely to provide a more accurate indication of the presence or absence of the influence of abnormal glucose metabolism.

Response: We thank the reviewer for this insightful methodological suggestion. We agree that HbA1c provides a more stable and accurate measure of long-term glucose metabolism compared to random blood glucose measurements, which may be influenced by acute illness, stress, and time of measurement. We have now included HbA1c as a covariate in all multivariable models(Page 14,”Model 3 adjusted for age, sex, race, heart rate, respiratory rate, MBP, potassium, chloride, bicarbonate, anion gap, BUN, creatinine, glucose, HbA1c…”) .To assess whether the prognostic value of PIV differs according to glycemic control, we performed subgroup analyses stratifying patients by clinically relevant HbA1c thresholds (<6.5% vs. ≥6.5%) (Page 20, Figure 5: Subgroup analysis for the effect of lnPIV on 28-day all-cause mortality.)

Comment 1.3: White blood cell counts are also significantly affected by the presence or absence of glucocorticoids , which are often administered to critically ill patients, but how was this factor taken into account in this analysis?

Response: We thank the reviewer for raising this important confounding factor. Glucocorticoid use is indeed common in critically ill patients and can significantly influence white blood cell counts, particularly neutrophils and lymphocytes, which are key components of the PIV calculation. To address this concern, we have included glucocorticoid use as a covariate in all fully adjusted models (Page 14,”Model 3 adjusted for …medications used.”). Glucocorticoid use was defined as administration of any systemic glucocorticoid (e.g., hydrocortisone, methylprednisolone, dexamethasone) during ICU stay, based on medication administration records. To assess whether the prognostic value of PIV differs between patients who received glucocorticoids and those who did not, we performed subgroup analyses stratifying patients by glucocorticoid use(Page 20, Figure 5: Subgroup analysis for the effect of lnPIV on 28-day all-cause mortality.). The attenuated effect in glucocorticoid users may be explained by the fact that glucocorticoids themselves alter white blood cell counts (inducing neutrophilia and lymphopenia), which may partially obscure the inflammatory signal captured by PIV (Page 21, line 21,”Glucocorticoids, often used for anti-inflammatory…”). Additionally, glucocorticoid use often indicates greater disease severity, where mortality risk may be driven by factors beyond immune-inflammatory status alone.

Comment 1.4: Looking at the AUC of the ROC analysis, it appears that simply using lymphocyte count as an indicator would be sufficient as a prognostic predictor without the need to use PIV, but it is necessary to clearly explain to readers the benefits of using PIV.

Response: We thank the reviewer for this critical and important question. We agree that lymphocyte count alone shows predictive value (AUC = 0.662), which is consistent with its central role in immune regulation(Supporting information,Page 4). However, to demonstrate the added value of PIV beyond single lymphocyte count, we have conducted comprehensive analyses including comparison with the SOFA score, integrated discrimination improvement (IDI), net reclassification improvement (NRI), and decision curve analysis. In the Discussion section (Page 22,line 10-17),we have expanded the explanation of why PIV offers advantages over single lymphocyte count.Below we detail our findings and revisions. Although the AUC values are similar, PIV captures a broader spectrum of the immune-inflammatory response by integrating neutrophils, monocytes, and platelets in addition to lymphocytes. To demonstrate clinical utility, we evaluated whether PIV provides incremental prognostic information beyond the established SOFA score, which is widely used in ICU settings. We compared two models: SOFA alone and SOFA + PIV(Supporting information,Page 5). Decision curve analysis was performed to assess the clinical utility of adding PIV to SOFA across a range of threshold probabilities. This analysis quantifies the net benefit of using a model for clinical decision-making by weighing true positives against false positives(Supporting information,Page 5,7).

---

\## Responses to Reviewer #2's Specific Comments

**Major Comments**

Comment 2.1-3: Hyperlipidemia is defined solely based on ICD-9/10 codes, without stratification by lipid subtype  (e.g., hypertriglyceridemia, hypercholesterolemia, mixed dyslipidemia). Given that different lipid abnormalities may have distinct inflammatory and prognostic implications, the authors should clarify which lipid phenotypes are primarily represented. If available, subgroup or sensitivity analyses based on triglyceride or cholesterol levels would substantially enhance the clinical relevance of the study.

Response: We thank the reviewer for this important methodological critique. We fully agree that different lipid subtypes may have distinct inflammatory and prognostic implications, and that stratification by lipid phenotype would enhance the clinical relevance of our findings. Using the multiply imputed datasets, we performed subgroup analyses stratifying patients by clinically relevant lipid thresholds:

**Triglycerides**: ≤150 mg/dL (1.7 mmol/L) vs. >150 mg/dL

**Total cholesterol**: ≤200 mg/dL (5.2 mmol/L) vs. >200 mg/dL

The results demonstrated that the association between lnPIV and 28-day mortality was consistent across all lipid subgroups(Page 20, Figure 5: Subgroup analysis for the effect of lnPIV on 28-day all-cause mortality.).

Regarding which lipid phenotypes are primarily represented in our cohort, based on the imputed lipid data:

Hypertriglyceridemia (TG >150 mg/dL), Hypercholesterolemia (TC >200 mg/dL), **Mixed dyslipidemia** (both TG >150 and TC >200).

Although we performed subgroup analyses based on triglyceride and cholesterol levels, the possibility of residual confounding from unmeasured lipid parameters (e.g., LDL-C, HDL-C, lipoprotein(a)) cannot be completely excluded. Future studies with complete lipid profiles are needed to further elucidate the relationship between specific lipid subtypes and PIV's prognostic value.

Comment 2.4-6:Although the authors adjusted for APS III, SOFA and SIRS scores, PIV is itself strongly correlated with disease severity. The possibility of residual confounding or overadjustment should be discussed more explicitly. Consider performing sensitivity analyses excluding severity scores or using alternative adjustment strategies to confirm the robustness of the association.

Response: We thank the reviewer for this important methodological critique. In our final revised models, we have taken a different approach: rather than adjusting for severity scores, we intentionally excluded them from Model 3 to avoid potential overadjustment, given that PIV itself is correlated with disease severity (Page 14,”Model 3 adjusted for …”). Instead, we evaluated the incremental value of PIV beyond severity by directly comparing SOFA alone vs. SOFA + PIV(Supporting information,Page 5).

Comment 2.7-9: The restricted cubic spline analysis identifies a threshold lnPIV value (≈5.15) beyond which mortality risk increases sharply. However, the clinical meaning of this threshold is not sufficiently discussed. The authors should translate this lnPIV value back to the original PIV scale and discuss how it could be applied in real-world clinical decision-making.

Response: We thank the reviewer for this valuable suggestion to enhance the clinical translatability of our findings. Identifying a clinically meaningful threshold is essential for translating statistical findings into practical risk stratification tools. We have addressed this concern through the following revisions. The threshold identified by restricted cubic spline analysis was lnPIV = 5.152. We have back-transformed this value to the original PIV scale using exponential transformation: **PIV ≈ e^5.152 = 172**. This threshold is now explicitly reported in Page 18,line 1. The threshold of PIV ≈ 172 has several important clinical implications: Patients with PIV values above 172 at ICU admission may be identified as high-risk and flagged for closer monitoring, more frequent clinical assessments, or consideration of early interventions.  However, we emphasize that this threshold requires external validation before clinical application, and should be interpreted in the context of other clinical parameters rather than as a standalone decision-making tool.

Comment 2.10-12:Although lnPIV showed a higher AUC than individual blood cell parameters, the overall discriminative ability remains modest (AUC ≈0.66). The manuscript should more cautiously interpret the predictive performance and clarify that PIV may complement, rather than replace, existing risk models. Comparison with established prognostic scores (e.g., SOFA alone vs. SOFA + PIV) would strengthen the argument for clinical utility.

Response: We thank the reviewer for these important observations regarding the appropriate interpretation of predictive performance and the need to position PIV as a complementary rather than replacement tool. We have addressed these concerns through several revisions throughout the manuscript. We agree with the reviewer that an AUC of 0.66 represents modest discriminative ability, and we have revised the manuscript to reflect this more cautiously. We have added explicit statements throughout the manuscript clarifying that PIV is intended to complement, not replace, existing risk models.(Page 18,line 5-13; Page 21,line 8-12;” ROC curve analysis showe...”). To strengthen the argument for clinical utility, we have directly compared the predictive performance of SOFA alone, PIV alone, and SOFA + PIV(Supplementary Figure S2).

Comment 2.13-14: The manuscript states that multiple imputation was used... but the imputation method, number of imputations and variables included are not described. These details should be clearly reported to ensure transparency and reproducibility.

Response: We thank the reviewer for emphasizing the importance of methodological transparency. We agree that complete reporting of multiple imputation procedures is essential for reproducibility. In response, we have substantially expanded the Statistical Analysis section to provide comprehensive details of our imputation approach. The following text has been added to the Statistical Analysis section: "For covariates with missing rates <20%, multiple…”(Page 8, line.15-21). To demonstrate the validity of our imputation approach, we added density plots comparing observed versus imputed values for key variables with missing data(Supplementary Figure S1).

Comment 2.15-16:The study is based on a single-center databasefrom a U.S. tertiary care hospital. The authors should more explicitly discuss the limitations regarding external validity, particularly for non-ICU settings and populations with different ethnic, socioeconomic, or healthcare characteristics.

Response: We thank the reviewer for this important reminder to explicitly address external validity. We agree that single-center studies require careful discussion of generalizability. We have substantially expanded the Limitations section to provide a more nuanced and comprehensive discussion of external validity(Page 24,line18-20,” Second, this was a single-center analysis…”)..

**Minor Comments**

Comment 2.17: Although MIMIC-IV studies are exempt from IRB approval, a brief ethics statement should be explicitly included in the Methods section, consistent with journal requirements.

Response: We thank the reviewer for this important reminder. We agree that all manuscripts should include a clear ethics statement, regardless of exemption status, to ensure transparency and compliance with journal requirements. We have added a comprehensive ethics statement to the Methods section(Page 5,line 5-10.) and Supporting information(Page 9).

Comment 2.18:Minor grammatical and stylistic issues are present (e.g., "imbalance lipid metabolism" should be "imbalance in lipid metabolism"). A thorough language edit would improve readability.

Response: We thank the reviewer for these specific language suggestions. We agree that clear and accurate language is essential for scientific communication.The example provided by the reviewer has been corrected throughout the manuscript.(Page 2,line 17,” an imbalance in lipid metabolism.”)

Comment 2.19:The manuscript alternates between "hospital mortality" and "in-hospital mortality." Please standardize terminology throughout the text and tables.

Response: We thank the reviewer for this careful observation. Consistent terminology is essential for clarity and professionalism in scientific writing. We have thoroughly reviewed the entire manuscript and standardized the terminology as suggested.(Page13-14, Table 2; Page15 Table 3)

Comment 2.20: Figure legends should be more descriptive, particularly for the restricted cubic spline and subgroup analyses, to allow interpretation without referring back to the main text.

Response: We thank the reviewer for this important suggestion. We agree that figure legends should be self-contained and allow readers to understand the key findings without constantly referring to the main text. We have thoroughly revised all figure legends to be more comprehensive and descriptive.

Comment 2.21: Units for laboratory values should be consistently reported across all tables.

Response: We thank the reviewer for this careful observation. Consistent reporting of units is essential for clarity and professionalism. We have thoroughly reviewed all tables to ensure that units are consistently reported throughout the manuscript. We have examined Table 1, Table 2, Table 3, and all Supplementary Tables to verify unit consistency.

Comment 2.22:The Discussion would benefit from a clearer comparison of the present findings with prior studies evaluating PIV or related inflammatory indices (e.g., NLR, PLR, SII) in critically ill or dyslipidemic populations.

Response: We thank the reviewer for this valuable suggestion. We agree that contextualizing our findings within the broader literature strengthens the discussion and helps readers understand the significance of our results. We have substantially expanded the Discussion section to include systematic comparisons with prior studies on PIV and related inflammatory markers in relevant populations(Page 22,line 6-9,” PIV encompasses a broader range of cell types compared to other inflammation-related indices (such as Platelet-to-Lymphocyte Ratio, Neutrophil-to-Lymphocyte Ratio, and Systemic Immune-Inflammation Index)…”). We have conducted direct head-to-head comparisons of multiple inflammatory markers within our own cohort to provide a comprehensive assessment of PIV's performance relative to other indices(Supplementary Table S3: Predictive performance of PIV and other inflammatory markers for in-hospital mortality).

---

\## Responses to Reviewer #3's Comments

Comment 3.1: In studies examining the Pan-Immune-Inflammation Value (PIV)—including observational research in hyperlipidemia patients—there is no single universally accepted "normal value." This is because PIV is a composite inflammatory index and its reference range depends on the study population and clinical context. Based on published observational and cardiovascular/metabolic studies: Author should need to incuat

Healthy individuals / low-risk populations:
≈ 200–400

Patients with metabolic disorders (including hyperlipidemia):
≈ 300–600

High inflammatory burden / poor prognosis groups:
> 600–800 (sometimes much higher depending on disease severity)

In hyperlipidemia cohorts specifically, many studies use:
Cutoff values should ideally be:

Derived from your study population

Adjusted for age, sex, comorbidities, and statin use

Most studies define “high PIV” using ROC analysis or percentile grouping, not standard laboratory reference ranges

Response: We thank the reviewer for this expert and highly valuable guidance on the interpretation of PIV values and thresholds. We fully agree that there is no universal "normal value" for PIV and that population-specific thresholds are essential. We derived our threshold directly from our study population using **restricted cubic spline (RCS) analysis**, which identifies the point at which the relationship between PIV and mortality changes significantly. This method is superior to arbitrary percentile-based cutoffs because it is driven by the outcome relationship rather than the distribution of the marker. Our substantially lower threshold (≈172) likely reflects the extreme physiological stress and immune dysregulation characteristic of critical illness, where even modest elevations in inflammatory markers carry significant prognostic implications. This finding underscores the critical importance of population-specific thresholds when interpreting PIV in clinical practice

Furthermore, this threshold was estimated from fully adjusted models that accounted for all relevant confounders, including age, sex, comorbidities (heart failure, atrial fibrillation, hypertension, diabetes, renal disease, etc.), and importantly, statin use, ensuring that the identified cutoff is independent of these factors. This population-specific, confounder-adjusted approach follows the reviewer's guidance and provides a clinically meaningful threshold for risk stratification in this particular population, while acknowledging that different populations may require their own derived cutoffs.

---

**Summary of Major Changes**

- Title revised to specify "critically ill patients"

- Added HbA1c and glucocorticoids as covariates; performed multiple imputation

- Identified PIV threshold ≈172; compared PIV with other inflammatory markers

- Demonstrated incremental value of PIV beyond SOFA (Table 5, Figure S2)

- Added 4 supplementary tables and 2 supplementary figures

- Publicly shared all analysis code on GitHub

We hope that these revisions have adequately addressed all concerns and that the manuscript is now suitable for publication in PLOS ONE.

Sincerely,

Xiao-Ying Huang, Xiang-Zheng

Corresponding author: syzx481@hbmu.edu.cn
